# Proteomic profiles comparison of three isolated bacteria strains from a copper processing area

**DOI:** 10.1186/1753-6561-8-S4-P197

**Published:** 2014-10-01

**Authors:** Louise Hase Gracioso, Ingrid Regina Avanzi, Marcela Do Passos Galluzzi Baltazar, Marcela Nunes Veiga, Luciana Jandelli Gimenes, Claudio Augusto Oller Do Nascimento, Elen Aquino Perpetuo

**Affiliations:** 1 Center for Environmental Research and Training, CEPEMA-POLI-USP; University of São Paulo, Biomedical Sciences Institute, ICB-USP, SP, Brazil

## Background

Release of toxic metals is one of most significant environmental problems since industrial revolution, mainly because heavy metals are not degraded and, therefore, remain in the environment. Some natural ecosystems may contain high heavy metals concentrations and, thus, it is not surprising that genes resistance to heavy metals are easily found in bacteria living in these environmental samples [1]. Soils, sediments and waters contaminated with xenobiotics compounds are suitable substrates for isolation of these adapted microorganisms [2]. Thereby, proteome analysis has become a powerful tool for investigating global changes in prokaryotic gene expression. Once the two-dimensional electrophoresis (2-DE) displays all bacterial soluble proteins expressed at specific culture conditions on gel, high throughput screening of these induced proteins is possible. This study has a purpose to isolated bacteria that tolerate high concentrations of copper and compared their proteomic profiles. The understanding about the copper metabolic role of these microorganisms it will be helpful to improve bioprocesses of decontamination.

## Methods

Microorganisms were isolated from mining wastes (samples of water and sediments) by culture enrichment technique; this procedure was repeated four times. Isolates were inoculated into MJS medium containing different concentrations of chloride copper (1mM, 2.5mM and 5mM) and incubated in plates for 72 h at 28°C. Strains have been identified by mass spectrometry equipment (MALDI-TOF) that produces a protein spectrum of each sample and then compares the obtained profile with a Biotyper data base. Cells were grown on MJS with and without copper (2.5 and 5 mM) incubated on an orbital shaker (150 rpm) at 28°C. The cells were collected in the exponential growth phase for extraction proteins procedure. Then, these citosolic proteins were analyzed using 2-DE technique, as described previously [3].

## Results and conclusions

Two bacteria strains were isolated from samples of sediment and one from water sample. Identification of strains isolated from sediment samples resulted in Pseudomonas aeruginosa and Acinetobacter pitii and from water sample also identified as Pseudomonas aeruginosa. Acinetobacter pitii and Pseudomonas aeruginosa (water sample) tolerated until 2.5 mM of copper and Pseudomonas aeruginosa from sediment sample tolerated 5 mM of copper. One study with Pseudomonas aeruginosa strain revealed this bacterium was able to tolerate only 0.77 mM of copper [4] and Andreazza et al., reported Acinetobacter sp. strain tolerated until 8 mM of copper. Pseudomonas aeruginosa and Acinetobacter pitii (from sediment samples) when grown up only in the MJS medium (without copper) expressed 206 and 247 proteins, respectively. Pseudomonas expressed 132 proteins when grown up in MJS with copper, where 38 were differentially expressed after copper exposure. Of the 188 proteins expressed by Acinetobacter (MJS with copper) 57 were expressed differentially. Pseudomonas aeruginosa (isolated from the water sample) reached the expression of 240 proteins (only in MJS medium). When grown up in MJS with copper, 80 proteins were expressed, where 65 proteins presented different expression after copper exposure. All these proteins may be involved in the resistance copper mechanism and the understanding of their metabolic role can be helpful for improve the bioremediation process.
